# Electromagnetic pulsed thermography for natural cracks inspection

**DOI:** 10.1038/srep42073

**Published:** 2017-02-07

**Authors:** Yunlai Gao, Gui Yun Tian, Ping Wang, Haitao Wang, Bin Gao, Wai Lok Woo, Kongjing Li

**Affiliations:** 1 School of Electrical and Electronic Engineering, Newcastle University, Newcastle upon Tyne, NE1 7RU, UK; 2 College of Automation Engineering, Nanjing University of Aeronautics and Astronautics, Nanjing 211106, China; 3 School of Automation Engineering, University of Electronics Science and Technology of China, Chengdu 611731, China

## Abstract

Emerging integrated sensing and monitoring of material degradation and cracks are increasingly required for characterizing the structural integrity and safety of infrastructure. However, most conventional nondestructive evaluation (NDE) methods are based on single modality sensing which is not adequate to evaluate structural integrity and natural cracks. This paper proposed electromagnetic pulsed thermography for fast and comprehensive defect characterization. It hybrids multiple physical phenomena i.e. magnetic flux leakage, induced eddy current and induction heating linking to physics as well as signal processing algorithms to provide abundant information of material properties and defects. New features are proposed using 1st derivation that reflects multiphysics spatial and temporal behaviors to enhance the detection of cracks with different orientations. Promising results that robust to lift-off changes and invariant features for artificial and natural cracks detection have been demonstrated that the proposed method significantly improves defect detectability. It opens up multiphysics sensing and integrated NDE with potential impact for natural understanding and better quantitative evaluation of natural cracks including stress corrosion crack (SCC) and rolling contact fatigue (RCF).

Emerging integrated techniques, sensing and monitoring materials degradation are increasingly required for characterizing the structural integrity and safety of infrastructure^1^,^2^. Nondestructive evaluation (NDE)^3^,^4^ is a typical effective sensing approach to recognize material characteristics and structural degradation without destroying the serviceability of a component or system. However, conventional NDE based on single modality sensing is not adequate to evaluate structural integrity with the required spatial resolution, coverage and accuracy^4^,^5^,^6^,^7^,^8^. A single NDE method using one modality of physics such as ultrasonic, electrical, magnetic, optical, thermal and radiography will not always be sufficient for the purpose of complete defects evaluation. To overcome the above problems, the use of multiple modality sensing and fusion^5^,^8^,^9^,^10^ in a complementary manner paths the way to enable a comprehensive understanding of material and structural characteristics. Integrating sensing techniques such as electromagnetic acoustic transducer (EMAT)^8^, infrared and optic thermography^9^, and magneto-optical visualization^10^ provide a promising multiphysics problem-solving approach to enhance the NDE performance for material and defect evaluation.

The sensing techniques based on the electromagnetic, i.e. eddy current^7^, magnetic flux leakage (MFL)^6^, and alternating current field measurement (ACFM)^11^ have been widely applied in NDE. Eddy current pulsed thermography (ECPT)^12^,^13^,^14^ is an emerging multiple modality NDE technique for conductive material which combines both advantages of pulsed eddy current (PEC)^7^ and infrared thermography^15^. The material properties such as electrical conductivity, magnetic permeability and thermal conductivity are used to identify and evaluate the features of interest. Material characteristics and structural stress, fatigue or damages are efficiently recorded and demonstrated through thermal image sequences can be evaluated by Joule heating via eddy current, heat conduction and infrared thermography^12^,^13^. Thermal patterns including the contrast against background of the tested object are analyzed by image processing and feature extraction e.g. differential absolute contrast (DAC)^16^, thermographic signal reconstruction (TSR)^12^, principle component thermography(PCT)^17^ and pulsed phase thermography (PPT)^18^ methods for defect characterization^12^,^14^,^19^. The superior performances of ECPT such as non-contact, high resolution, fast detection in a large area with rich transient information enable extensive research and NDE applications in recent years^14^,^15^. Wilson *et al*.^15^ proposed the PEC thermography to detect multiple cracks of rail rolling contact fatigue (RCF). Cheng^20^ and He^21^ ‘lighted’ and detected impact damages of composite material using ECPT. He *et al*.^22^ investigated the ECPT for detection of corrosion blister in mild steel. Li *et al*.^23^ used the ECPT for bond wire state detection in electrical modules. Tian *et al*.^24^ applied the ECPT for early fatigue evaluation of gear. Yin *et al*.^13^ reported the physical interpretation of ECPT and discussed the links between mathematical and physical models. Gao *et al*.^14^ extracted spatial and time patterns for automatic NDE based on the ECPT transient thermal sequences. However, the problems of in-homogenous heating, limited heating area and blocking effect of coil^24^,^25^,^26^ in reflection mode ECPT are still challenging the accurate material and defect quantitative characterization. Lahiri *et al*.^27^ reported low frequency alternating magnetic field for thermographic NDE of defect with large area. Jäckel *et al*.^28^ proposed an electromagnet yoke for external magnetic field to enhance the crack detection contrast by induction thermography. Hansen *et al*.^29^ designed an asymmetric induction coil for generating uniform heating. Netzelmann *et al*.^30^ developed a measurement system with induction generator scanning for rail surface defects inspection at different speed up to 15 km/h. Shepard *et al*.^31^ described advances and the analysis of pulsed thermographic data for defect detection with increased spatial and temporal resolution. The implementation of uniform heating in a large area together with a wide open-view region for defect imaging is required to overcome the above problems of ECPT. To enhance the NDE performance of the ECPT, more physics integration including but not limited to the eddy current with thermal imaging are urgently required for comprehensive NDE of natural multiple defects, larger area detection of structures with free-form surface and evaluations of carbon fiber reinforced plastics (CFRP) flaws^20^,^21^ and RCFs^25^ etc.

In this work, we developed an electromagnetic pulsed thermography using two physical phenomena of magnetic field and eddy current for induced heating for fast and comprehensive defect characterization. The response signals are interpreted to provide abundant material and defective information through temporal and spatial responses. The proposed method with multiphysics sensing and interpretation are based on the integration responses of different NDE techniques, e.g. MFL or EC, and a fusion of different physical phenomena for heating, these includes (1) induced eddy current generates Joule heating, (2) alternating magnetization/demagnetization produce hysteresis loss for heating, and (3) leakage magnetic flux with stray loss for different time and amplitude responses^32^. The thermal patterns captured by infrared imaging enables the visualization of eddy current, magnetic field behaviors and their heating effect. In addition, the new feature of 1^st^ derivation thermal pattern calculation is proposed for enhancing natural cracks inspection. The system operational principle is examined in simulation with interactions of multiple physical field behavior. New features are extracted and confirmed in experiments to estimate crack orientations through the response time and speed, which reflect different percentage contributions of magnetic flux leakage and eddy current for heating as well as defect evaluation. Different spatial and temporal responses of multiphysics reveal the promising relative uniform field excitation together with an open-view imaging for accurate defect detection and characterization. The advanced performance of the proposed method e.g. robust to lift-off changes and effective for defect orientation and depth estimation opens up multiphysics sensing and integrated NDE with potential significant impact for fast quantitative evaluation.

## Methods

### Implementation of electromagnetic pulsed thermography system

**Figure 1:**
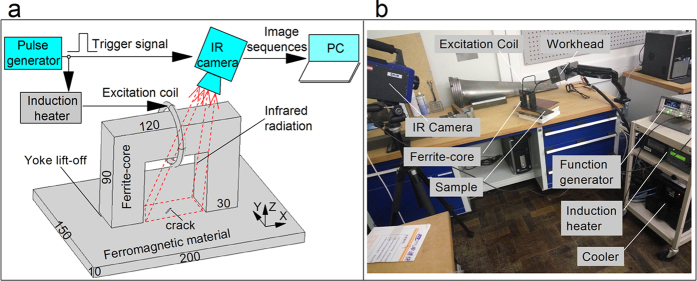
Fabrication of electromagnetic pulsed thermography system. In (**a**), the schematic diagram with well-established excitation and sensing configuration is depicted. The U-shaped ferrite-core act as inductor; the distance between the pole shoes and the ferromagnetic test object is defined as yoke lift-off. The size of the ferrite-core and the sample are also illustrated for further multiphysics modeling, simulation and experimental arrangement. In (**b**), the experimental system is presented to show further capabilities of the approach for material properties and defect characterization (Details of operation are provided below).

To implement the multiphysics sensing approach for comprehensive NDE, a new electromagnetic pulsed thermography system is illustrated with a schematic diagram in Fig. 1a and b. The proposed system is based on the comparison studies of the magnetic flux leakage and ECPT techniques and combination of both advantages. A high power and high frequency alternating electrical current is generated by a pulse generator and induction heater to drive the excitation coil. A very intense and rapidly changing magnetic flux is produced in the space within the coil. Compared with the previous ECPT^13^,^15^, the most significant improvement of this procedure is using a ferrite-core to concentrate most of the magnetic flux into a magnetic circuit and guiding them to flow into the test object with a broad and uniform field distribution. In addition, the use of a ferrite-core (magnet yoke) enables the decrease of magnetic resistance in the magnetic circuit and enhances the magnetic flux intensity in the ferromagnetic test object for efficient induction heating. Moreover, the local region between two pole shoes of the magnet yoke provides a wide open-view area for defect detection and visualization by full coverage infrared imaging, which will significantly benefit for quantitative NDE. Thermal image sequences including multiphysics spatial and temporal responses and defect characteristics are transmitted to a computer for signal processing and defect features extraction.

The characteristics of the proposed electromagnetic pulsed thermography system enable the integration of multiple physical phenomena for material and defect characterization. These includes: (i) Magnetic flux leakage with stray loss: Any geometrical discontinuity or local anomalies existing in the measured area lead to the magnetic flux leakage to air around the defective area due to the abrupt changes of magnetic permeability, which is the MFL principle^6^,^33^. Around the defective area, the local magnetic field distribution inside of the test object will be disrupted and represented as high/low magnetic flux density. Based on the stray loss due to the leakage of magnetic flux, this paper uses remaining magnetic field inside of test object around the defective area to induce different eddy current and inductive heating responses for defect detection. (ii) Induced eddy current: significant amount of eddy currents will be induced by rapidly alternating magnetic field and orthogonally distributed against the magnetic field lines in the surface and subsurface region of test object^13^,^24^. Similar to the MFL, the features of interest manifested as an abrupt change of electrical conductivity will lead to the disturbance of eddy current field and represent as increased/decreased density around the defective area. Based on the eddy current loss, the heating effect of eddy current is utilized for the visualization of the eddy current field distribution and intensity behaviors for the defect detection. (iii) Induction heating with multiphysics^34^,^35^: The induced eddy current will give rise to local resistive heat by Joule heating^13^. Due to the hysteresis effect^36^,^37^ of the ferromagnetic material, it naturally offers resistance to intense alternating magnetic field because of the repeatedly magnetize/demagnetize processes. The rapid periodic creation and annihilation of magnetic domains by domain wall movements^38^,^39^ cause considerable internal friction damping, magnetostrictively moved dislocation and heating inside the material, which is potential for speedy and efficient thermographic NDE due to the secondary source of heat^40^,^41^. Additionally, the leakage of magnetic flux with stray loss also induce different inductive heating response due to magnetic field distribution and intensity variation inside of test object. Above all, the magnetic flux and eddy current field diversion and intensity changes around the defective area will lead to the different heat density and thermal contrast between defective and defect-free area, which makes defect visible using an IR camera. The physical field distribution and dynamic behaviors of magnetic field and eddy current field are lighted by the above heating effect and visualized through thermal imaging. The dynamic thermal behavior also can be used to characterize defective features because of the heat conduction and material thermal conductivity variation^13^,^24^. When the proposed system is installed on an inspection car which is rapidly driving on the rail, the permanently electromagnetic pulsed heating and thermal imaging are continually working for natural cracks location and characterization.

### Multiphysics modelling and simulations

In this section, the interactions behavior of the multiple physical field by using multiphysics modeling and simulations conducted with COMSOL Multiphysics 4.3b^6^,^25^ is conducted. A three-dimensional (3D) model is built on account of the proposed system configuration in Fig. 1a. The radius and wire diameter of the excitation coil are 25 mm and 6.35 mm, respectively. The dimensions of the U-shaped ferrite-core are 120 × 30 × 90 mm^3^ in length, width and height with a vacant region of 60 × 30 × 60 mm^3^ between two pole shoes of the ferrite-core. The steel sample with 200 × 150 × 10 mm^3^ includes three oriented slots with the orientations 0 degree, 45 degree, 90 degree and dimensions of 10 × 1 × 5 mm^3^ in length, width and depth, respectively. The distance between the pole shoes of the U-shaped ferrite-core and the ferromagnetic test object is 1 mm, which is defined as yoke lift-off. The materials properties of the ferrite-core, steel and air are setup based on their real parameters such as relative permeability 5000, 800, 1; electrical conductivity (unit S/m) 0.01, 4.03 × 10^6^, 0.01 and thermal conductivity (unit W/(m × k)) 2.90, 44.50, 2.57 × 10^−2^. The fine mesh was generated within the problem regions of interest, e.g. three slots areas. The mesh quality was improved in the steel sample to achieve accurate multiple physical fields distribution without too much sacrifice of computing time. The entire 3D model was divided into 215571 tetrahedral elements for the finite element calculation. The calculation is implemented based on the AC/DC module of COMSOL with the maximum number of iterations 10. The simulation of 3D model took about 28 minutes in a typical 2.2 GHz Intel Core i5 processor computer with 8 G memory. Electrical current 380 A with frequency 256 kHz is driven into the excitation coil to generate intense alternating magnetic flux in the space within the coil. Most of the magnetic flux are concentrated in the ferrite-core and guided into the test object for inducing eddy current and heating in order to identify defective characteristics. Duration of induction heating is setup with 300 ms, which is enough to generate effective thermal response and contrast for material properties and defect characterization.

### Experimental set-up and samples

The experimental system of the proposed electromagnetic pulsed thermography is developed as shown in Fig. 1b. It consists of a function generator, heating source devices, excitation coil, ferrite-core, test sample and an IR camera as well as a PC for signal processing. A function generator Agilent 33500B is used to produce a pulsed signal to trigger and control the operation of the IR camera and heating devices. A precision induction heating device, Easyheat 224 from Cheltenham Induction Heating, Ltd., together with a cooler and a workhead, is used for coil excitation, with a maximum excitation power of 2.4 kW, a maximum current of 400 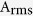, and an excitation frequency range of 150–400 kHz (380 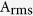 and 256 kHz are used in this study). The excitation coil is made of 6.35 mm diameter high-conductivity hollow copper tube where water cooling is implemented to counteract direct heating of the coil. To fit the U-shaped ferrite-core structure, the coil is designed as a rectangular shape and wound on the magnet yoke. The ferrite-core is made of MnZn ferrite material with parameters and sizes identical to simulation.

A mild steel sample^13^ containing a narrow, surface breaking slot in length 30 mm, width 0.5 mm and depth 6 mm is employed in the experimental tests. Through changing the relative position between ferrite-core and sample, various crack orientations to the excitation are detected and visualized by an IR camera. A short piece of rail track sample including rolling contact fatigue with natural multiple cracks is also employed for test. For defect depth estimation, three pieces of steel sample with 5 mm thickness, 150 mm length and 50 mm width are employed for test. Three slots of which the depths are 2 mm, 3 mm and 4 mm, respectively, with same defect length 10 mm and width 1 mm. The state-of-the-art IR system Flir SC7500 is used to record the temperature change and thermodynamics behavior, which is a Stirling cooled camera with a 320 × 256 array of 1.5–5 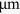 InSb detector. It has a sensitivity of <20 *mk* and a maximum full frame rate of 383 Hz with the option to increase the frame rate due to windowing of the image. In this study, the frame rate is 1253 Hz with a 160 × 128 windowing array. The same heating duration to simulation is used for experimental tests. Two seconds videos are recorded by the IR camera, and the obtained thermal image sequences are transmitted to the PC where the features extractions are implemented.

## Results

### Multiphysics sensing and integration principle - interpretation

**Figure 2:**
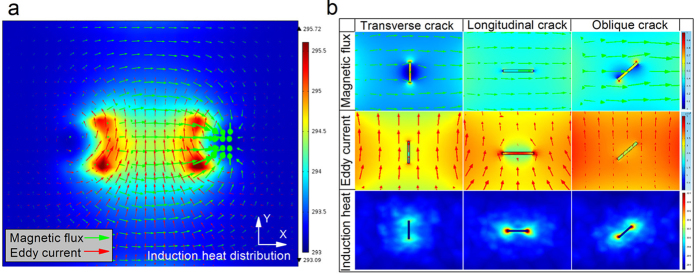
Simulation results of multiple physical field interactions behavior. In (**a**), the stimulated magnetic flux vectors (green) and induced eddy current vectors (red) in the measured area are depicted together with induction heat distribution. In (**b**), three physical field interactions behavior to transversal, longitudinal and oblique oriented cracks are illustrated to characterize the defect.

A simulation study of multiphysics sensing is carried out to examine the behavior and the integrated sensing principle of multiple physical field interactions. These results are illustrated in Fig. 2. From the results of the defect-free sample in Fig. 2a, it is apparent that a relative uniform distribution of magnetic field, eddy current and induction heat are clearly presented in the tested object between the pole shoes of the magnet yoke. Magnetic flux flows in the sample, as horizontal (green) arrows. Eddy current is induced by the alternating magnetic flux and flows in the region of interest with a distribution orthogonal to the magnetic field, shown as vertical (red) arrows. Based on the two heating effects^35^,^37^,^40^ and the distribution of the orthogonal field, it can be seen that the induction heat is correlated with the crack directions. In particular, eddy current provides little heat if the crack direction is parallel to the eddy current field. On the other hand, there is no distinct hysteresis heat when the crack direction is located parallelly to the magnetic flux vectors direction. The magnetic flux and eddy current distribute with higher intensity around the internal borders and corners of the ferrite-core poles to produce local hot-spot areas with higher heat density.

With the uniform field excitation, the distribution of three physical fields and the result of their interaction with a crack (slot) with different orientation to the magnetic field direction are illustrated in Fig. 2b. It represents different responses of magnetic field and eddy current to transversal and longitudinal oriented crack for heating. A transversal crack produces the greatest magnetic flux leakage into air and creates the largest disruption of the magnetic field within the test object due to their perpendicular orientation and stray loss of leakage magnetic flux. It results in the higher magnetic flux density around the crack tips and lower density in the middle of crack. On the other hand, the induced eddy current flow around the longitudinal crack is disrupted due to their perpendicular orientation to each other. The branching of eddy current results in higher density around the crack tips and lower density in the middle of the crack. Additionally, there are no significant eddy current field changes around the transversal crack and no distinct magnetic field variation around the longitudinal crack. Hence, the defective information of transversal and longitudinal cracks is mainly manifested by the diversity of the field direction and intensity changes of magnetic field and eddy current, respectively, which is shown as different inductive heating patterns. It also can be seen that thermal contrast around the longitudinal crack is higher than in case of the transversal crack as well as an unsymmetrical distribution of them around the oblique crack. It is dependent on the energy distribution that different percentage contributions of magnetic field and eddy current for heating and their orthogonal field distribution interact to different oriented defects. The interactions behaviors of the above multiple physics for heating are promising to produce more comprehensive information of material to design new features for defect inspection based on the thermal responses documented in images with information in space and in time.

### Invariant feature response to crack orientations

**Figure 3:**
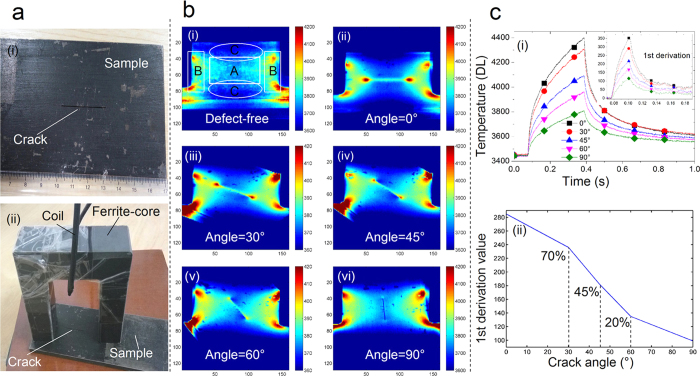
Experimental results and features for different oriented artificial cracks. In (**a**), the steel sample with a surface breaking slot in defect length 30 mm, width 0.5 mm and depth 6 mm (i) and the inductor with the excitation coil and the U-shaped ferrite-core (ii) are depicted. Different crack orientation can be obtained by rotating the ferrite-core and the sample direction relatively. In (**b**), thermal patterns are illustrated in the defect-free sample (i) and in samples including different oriented cracks with orientations from 0 degree to 90 degree (ii–vi). In (**c**), (i) illustrates the 1^st^ derivation of the thermal transient responses, (ii) the invariant feature – the 1^st^ derivation value at the start of pulse excitation has illustrated a 98% correlation with the orientations of the artificial cracks.

**Figure 4:**
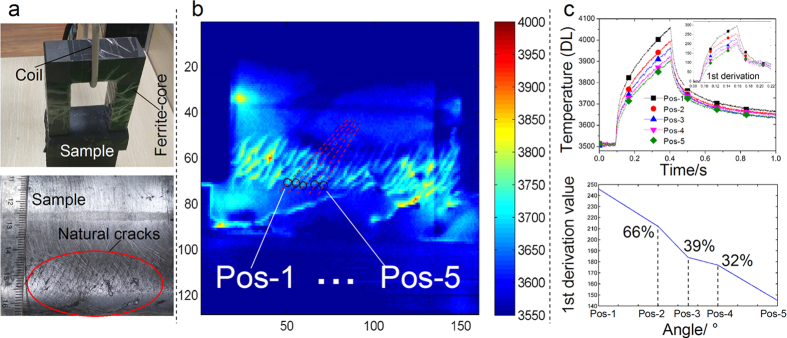
Experimental results and features for natural cracks. In (**a**), the rail sample with natural cracks (i) and the excitation are depicted; the black painting on the sample is to reduce the influence of the surface emissivity. In (**b**), the thermal patterns are well reflecting the natural cracks profile compared with the optical imaging in Fig. 4a; the different oriented cracks are artificially selected based on thermal contrast as well as the crack tips positions of Pos-1 to Pos-5. In (**c**), the thermal transient responses to different oriented cracks and their 1st derivation are depicted. The response to natural crack orientations vs the 1^st^ derivative feature reveals a good degree of correlation (98.3%).

A new feature is applied for defect characterization based on the above multiphysics sensing and integration principle. After pre-processing of the thermal images with smoothing and noise reduction^24^,^42^, by applying the new developed feature, the 1st derivative of the thermal response at the crack tips is extracted. It represents the thermal rising speed and has a relationship to the response speed of the interaction of the magnetic flux and the eddy currents with the crack. It has been applied to identify different orientation of artificial and natural cracks as confirmed experimentally in Figs 3 and 4. As shown in Fig. 3b(i), there is a relative uniform heat distribution in region A of the defect-free sample together with four hot-spot areas in region B due to the diffusion of the magnetic field lines and the eddy current distribution. The proposed feature applied for identifying crack orientations is invariant to the defect location e.g. no matter in the regions of A and B. The open-view thermal imaging here without coil block^13^ of the conventional ECPT^6^ together with uniform field heating excitation enables the proposed system for potential comprehensive defect visualization and quantitative characterization. As shown in Fig. 3b(ii–vi), artificial cracks at five orientations have been successfully indicated by the thermal patterns and contrast. The temperature around crack tips is decreasing when crack directions are changing from the orientation 0 degree to 90 degree as shown in Fig. 3b(ii–vi). In Fig. 3c(i), the slopes and amplitudes of thermal transient responses indicate clear relationship to cracks orientations. To quantify how well the relationship between the developed new feature and crack orientations, several random experimental tests have been carried out based on the Fig. 3 to obtain two random variables that the 1st derivation features and crack angles. A Pearson correlation coefficient of the above two random variables is calculated in the Matlab to measure their linear dependence. The new 1^st^ derivation features extracted from thermal transient responses demonstrate a good degree of correlation with the different orientations of the artificial cracks as shown in Fig. 3c. It shows promising results that the invariant 1st derivation features are correlated with crack orientations with 98% for defect quantitative characterization. It is through the different percentage contributions of the induced eddy current and magnetic flux leakage to heat response time and speed. In addition, it also has been applied to natural multiple cracks of rolling contact fatigue (RCF) in railway^15^,^25^ as illustrated in Fig. 4a–c, which present a full coverage high resolution imaging of the natural defects map. The developed 1st derivation features also reflect good correlation with the selected natural crack orientations, which shows good consistency to Fig. 3. The above results demonstrate the robust capability of the proposed system for defect orientation assessment and quantitative characterization with full coverage defective area visualization. Similar to artificial cracks, Fig. 4c also indicates the promising results of 98.3% correlation of the invariant feature with natural crack orientations. It is understood that the calculation of the temporal derivative of a measurement signal significantly increases the measurement noise. The usual way is to perform first a smoothing or a polynomial fitting to measurement data^42^, and then calculate the 1st or the 2nd derivative^12^,^42^ and shape of the signals such as principal components analysis (PCA) and independent component analysis (ICA)^13^,^20^. Further features can be extracted from the thermal transient responses^24^ using the proposed electromagnetic pulsed thermography system and the physics-based modeling and pattern mining techniques^32^ for quantitative NDE of the multiple and (tiny) natural cracks.

### Evaluation of lift-off influence and defect depth

**Figure 5:**
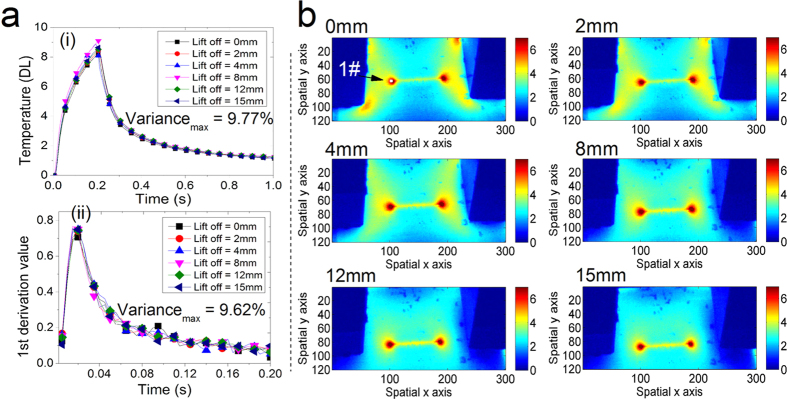
Experimental results with robust features to lift-off variation. In (**a**), the temperatures amplitude around crack tip position 1# with 9 pixel points average (i) and related 1^st^ derivation value (ii) with maximum variation about 9.77% and 9.62%, respectively, present robust to yoke lift-off variation range from 0 mm to 15 mm. In (**b**), thermal patterns of a transversal defect with different yoke lift-off values are illustrated that better heating uniformity is obtained.

To evaluate lift-off influence, experimental studies are conducted by changing the distance between the pole shoes of the U-shaped ferrite-core and the ferromagnetic test object. The yoke lift-offs are varied ranging from 0 mm to 15 mm with a step of 1 mm, which is controlled and measured by a vernier caliper device. The sample same to Fig. 3 with defect orientation 0 degree is used for the results analysis as shown in Fig. 5. The temperature amplitude in the selected local area 1# around the crack tip position with 9 pixel points average are illustrated in Fig. 5a(i) as well as the related 1^st^ derivation feature in Fig. 5a(ii). The variances of the temperature amplitude and the 1^st^ derivation feature in different lift-offs conditions are calculated to estimate the robustness of the proposed system on lift-offs changes. The maximum variance of the temperature response to different lift-offs in experiments is 9.8%, and that value of the 1^st^ derivation feature is 9.6%, as illustrated in Fig. 5a. The increase of the lift-off causes better heating uniformity as shown the thermal patterns in Fig. 5b. Overall, the above results illustrate the proposed system is robust to lift-off variation if the features temperature amplitude and 1^st^ derivation value are used together for defect characterization.

**Figure 6:**
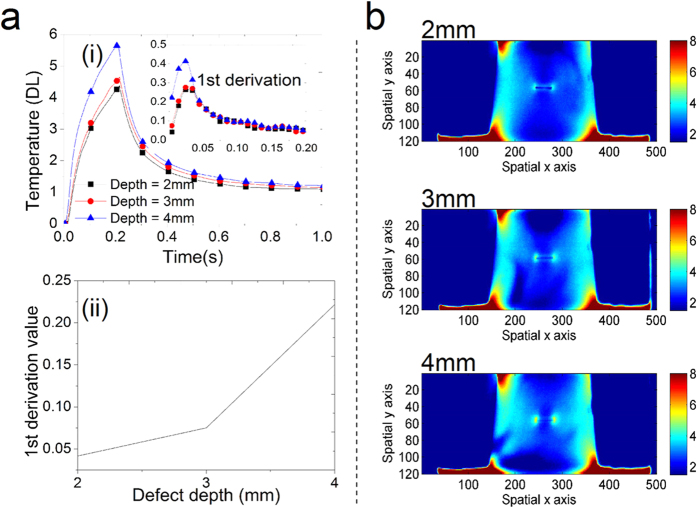
Experimental results for defect depth estimation with yoke lift-off 4 mm. In (**a**), the features of temperature amplitudes around cracks tip position with 9 pixel points average and related 1^st^ derivation value validate that defect depth can be identified. In (**b**), different thermal patterns are illustrated response to three different defect depth 2 mm, 3 mm, 4 mm of steel sample.

In order to further estimate defect depth, experimental studies are also implemented using the proposed electromagnetic pulsed thermography system with yoke lift-off 4 mm. Steel samples including three different defect depth 2 mm, 3 mm and 4 mm are used. Sample thickness is 5 mm and material properties are same to previous test objects in Figs 3 and 5. From Fig. 6a, it can be seen that the temperature amplitudes around crack tip position and the related 1^st^ derivation value can be used to identify defect depth^43^,^44^. The defect depth information is reflected by the above two thermal features due to the response speed/time of the magnetic flux and the eddy currents with cracks for heating. Thermal patterns at the end of heating are illustrated in Fig. 6b for defect depth estimation. The multiphysics behavior e.g. eddy current or thermal field distribution and intensity variation including optical flow pattern^24^ can be applied for defect classification and quantification^32^,^45^ and their uncertainty evaluation^43^,^45^, which will be further investigated and published.

## Discussion

The work has demonstrated a novel electromagnetic pulsed thermography with interpretation of multiphysics sensing and invariant features extraction for fast natural cracks inspection and quantitative characterization. The integration of multiple physical phenomena namely magnetic flux leakage with stray loss, induced eddy current and induction heating and their interactions behavior have been shown and analyzed with multiphysics spatial and temporal responses in simulation and experiments. The stimulated relative uniform field excitation together with an open-view thermal imaging to characterize artificial slot at different orientations and open/closed natural multiple cracks have been discussed. The response speed and time features due to different percentage contributions of magnetic flux leakage and eddy current to heat have been analyzed for efficient induction heating and fast quantitative NDE. The research contributions can be drawn as follows: (i) the proposed approach and system provide a relative uniform field excitation and enlarges the heating area and efficiency by using a ferrite-core, which benefits quantitative defect characterization; (ii) a wide open-view thermal imaging region is produced in the measured area accompanied with uniform field heating excitation for defect visualization, which is potential applied for NDE of complex free-form surface test object; (iii) the proposed system reveals a robust combined magnetic flux leakage and eddy current with different speed and time responding features/signatures of multiphysics linking to crack orientations as different percentage contributions to the heating; (iv) invariant feature can be extracted from the multiple physics system e.g. response time in combination of the eddy current and magnetic flux leakage responses. The developed new 1^st^ derivation feature demonstrates robust capability for crack orientations assessment in comparison with conventional systems and data fusion^13^,^14^; (v) the proposed method is robust to lift-off changes and effective for defect depth estimation with proposed 1^st^ derivation and thermal pattern features; (vi) the early time responses e.g. several milliseconds, and their integrative features of multiphysics can speed up quantitative NDE for not only artificial crack (open cracks) but also multiple natural cracks (short cracks). This work provides timely approach for imaging and visualizing tiny cracks (short, natural cracks) including stress corrosion crack (SCC) and rolling contact fatigue (RCF). It has potential to bridge the gaps of micro and marco non-destructive testing and evaluation. It can also extend the approach for *in-situ* scanning inspection with different speed and structural health monitoring.

## Conclusions and Future Works

This paper proposes an electromagnetic pulsed thermography for natural cracks detection and characterization. The key character of this method applies two physical phenomena of magnetic field and eddy current for induced heating, fast and comprehensive defect characterization. Based on simulation and experimental studies, invariant features response to defect orientations are provided with the interpretation of multiphysics sensing and integration phenomena. It is robust to lift-off changes and effective for defect depth estimation. The open-view thermal imaging and relative uniform heating generation are benefit for defect quantitative detection and characterization. This work paths the way to identify natural defects e.g. SCC or RCF for future QNDE and *in-situ* applications for guidance of maintenance, lifetime prediction and extension.

The coupling and separation of multiphysics with multiple heating effects of hysteresis, eddy current losses, surface heating flow characteristics^24^ with surface quality e.g. emissivity^46^, roughness and texture^47^ lead to the future multiple functional evaluation of material properties and defective characteristics. This work will open up multiphysics sensing and integrative fast imaging systems for comprehensive and quantitative NDE. More experimental studies on sensing and imaging, and analysis for better understanding the multiphysics interactions behavior, their modeling and quantitative validation for different test objects, defect depth and natural defects will be undertaken in future works. As previous studies concentrate on hysteresis loss and eddy current loss amplitudes, but ignore of their response speed, more studies on multiple physics under different transient and scales will be undertakes to develop physics-mathematical pattern modeling and mining for sensing, imaging and evaluation of natural cracks. It is important for future research on crack initiation and lifetime prediction and extension.

## Additional Information

**How to cite this article**: Gao, Y. *et al*. Electromagnetic pulsed thermography for natural cracks inspection. *Sci. Rep.* **7**, 42073; doi: 10.1038/srep42073 (2017).

**Publisher's note:** Springer Nature remains neutral with regard to jurisdictional claims in published maps and institutional affiliations.
